# PrimerSNP: a web tool for whole-genome selection of allele-specific and common primers of phylogenetically-related bacterial genomic sequences

**DOI:** 10.1186/1471-2180-8-185

**Published:** 2008-10-20

**Authors:** Jiqiang Yao, Hong Lin, Allen Van Deynze, Harshavardhan Doddapaneni, Martha Francis, Eliana Gertrudes Macedo Lemos, Edwin L Civerolo

**Affiliations:** 1 Citrus Research Board, 323 W. Oak Street, Visalia, CA 93291, USA; 2 USDA-ARS, San Joaquin Valley Agricultural Science Center, Parlier, CA 93648, USA; 3 Seed Biotechnology Center, University of California, One Shields Ave, Davis, CA 95616, USA; 4 University of California Davis, Department of Viticulture and Enology, Davis, CA 95616, USA; 5 University of California, Department of Plant Pathology, CA 95616, USA; 6 Departamento de Tecnologia, Faculdade de Ciências Agrárias e Veterinárias, Universidade Estadual Paulista, Câmpus Jaboticabal, Via de Acesso Prof. Paulo Donato Castellane, s/n, km 5, 14884-900 Jaboticabal, São Paulo, Brasil

## Abstract

**Background:**

The increasing number of genomic sequences of bacteria makes it possible to select unique SNPs of a particular strain/species at the whole genome level and thus design specific primers based on the SNPs. The high similarity of genomic sequences among phylogenetically-related bacteria requires the identification of the few loci in the genome that can serve as unique markers for strain differentiation. PrimerSNP attempts to identify reliable strain-specific markers, on which specific primers are designed for pathogen detection purpose.

**Results:**

PrimerSNP is an online tool to design primers based on strain specific SNPs for multiple strains/species of microorganisms at the whole genome level. The allele-specific primers could distinguish query sequences of one strain from other homologous sequences by standard PCR reaction. Additionally, PrimerSNP provides a feature for designing common primers that can amplify all the homologous sequences of multiple strains/species of microorganisms. PrimerSNP is freely available at .

**Conclusion:**

PrimerSNP is a high-throughput specific primer generation tool for the differentiation of phylogenetically-related strains/species. Experimental validation showed that this software had a successful prediction rate of 80.4 – 100% for strain specific primer design.

## Background

Clinical and environmental microbiology often requires the detection of a particular bacterial strain with/without the presence of other closely related strains/species in the samples [1]. Standard PCR is by far the most efficient method in pathogen detection and disease epidemiology research. The critical part of this method is the specific primer design. The challenge of the primer design is that PCR primers are being designed to amplify from one sequence, but not from other homologous sequences. The general approach of specific primer design by biologists is often performed manually by searching primer sequences against a DNA database, followed by experimental tests. However, Single Nucleotide Polymorphisms (SNPs), which represent micro but stable DNA variations, are difficult to analyze through regular BLAST searches and are often ignored while designing specific primers. Further, manually designing genome-wide primers based on sequence alignments can be challenging if strains have low sequence variability. An effective approach to process such a large amount of genomic data requires computational tools that are capable of processing large amounts of data and yet are easy to handle.

Although a large number of primer design programs are available, very few packages are available for allele-specific primer design. Primer Premier from Premier Biosoft International has an allele-specific PCR primer design function but it is a commercial package which is not free to the public. Primo [2], Primique [3] and Amplicon [4] are both free to the public but they require either user-defined homologous genes or sequence alignment files. All these software do not support batch design for multiple query sequences of a particular strain/species. None of these programs take into consideration the whole genome sequences of the non-target strains when designing specific primers. Therefore, the primers' specificity needs to be further improved before they can be used for detection.

In this paper, we describe a new software program, PrimerSNP, which designs strain-specific primers that can specifically amplify the genomic sequences of a particular bacterium strain but not the genomic sequences of other phylogenetically-related strains. It automatically identifies unique SNPs of the strain and design primers based on these unique SNPs. It also quantifies the primer specificity by calculating a weight score, which takes into account both the number and location of SNPs on the primers.

Although PrimerSNP is initially developed to design specific primers, a second function is also added, which designs common primers that could amplify all the homologous genes. Similar programs for common primer design exist such as GeneFisher [5], but this program does not support the batch design of primers.

Compared to currently available software, PrimerSNP reduces users' involvement to a minimum level: only uploading of multiple genomic sequences is required. It can automatically search for unique SNPs from the input genomic sequences. Importantly, this software has a high successful prediction rate (>80%) as validated by our experiments.

For user's convenience, a windows version of this software is prepared and can be downloaded from the main page.

## Implementation

**Figure 1:**
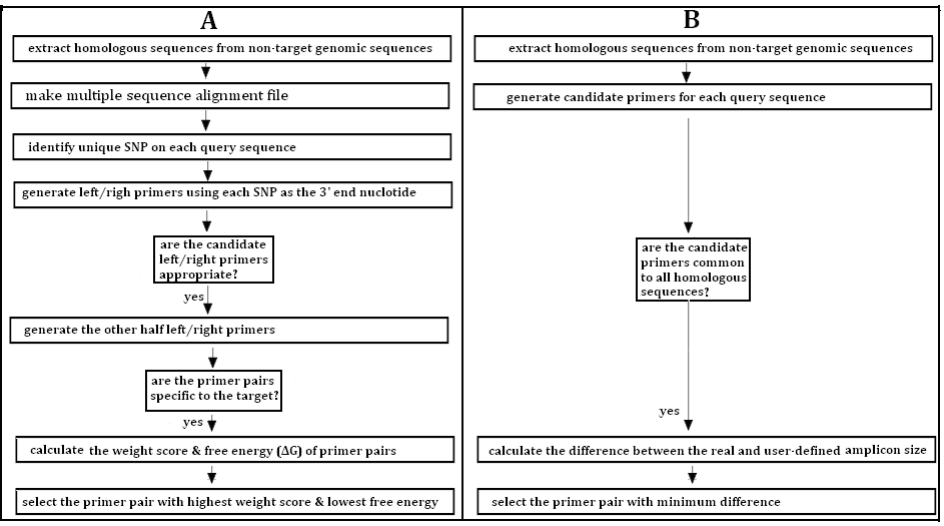
**The flowchart of the implementation of specific and common primer design**. (A) specific primer design (SPEC). (B) common primer design (COMN)

PrimerSNP is built around other public domain programs, such as BLAST [6], ClustalW2 [7], me-PCR [8] and Primer3 [9]. Implementation of PrimerSNP is shown in Figure 1. Panel A shows the flowchart of specific primer design (SPEC) and panel B shows the flowchart of common primer design (COMN).

**Figure 2:**
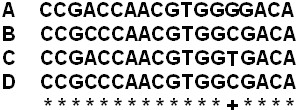
**A schematic representation of a unique SNP**. A is the query sequence; B, C and D are the reference sequences; '*' indicates identical nucleotides; '+' indicates the location of a unique SNP. 'G' is a unique SNP of the query sequence A.

**Figure 3:**
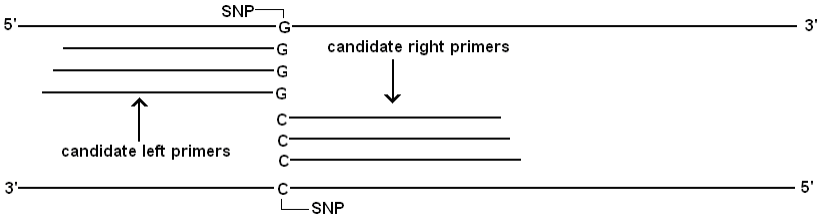
**Generation of candidate left and right primers**. A series of fragments with a single nucleotide difference of user-defined length to the left or right of each SNP are selected as candidate left or right primers.

**Figure 4:**
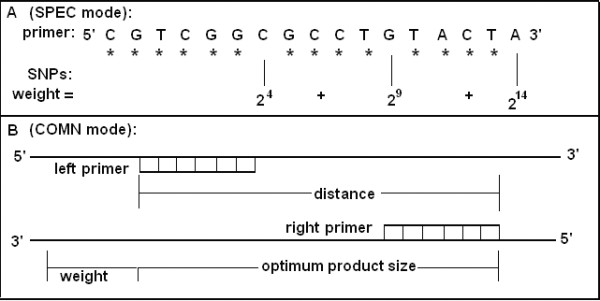
**Primer weight score calculation**. (A) SPEC mode: The first 15 nucleotides starting from the 3' end are used for the calculation. A value of 2^14 ^is assigned to the first nucleotide, 2^13 ^to the second and so on if that nucleotide is a SNP. Primer weight score is the sum of all the values assigned to SNPs. (B) COMN mode: primer weight is the difference between the optimum amplicon size and the actual amplicon size (default = 600 bp).

**Figure 5:**
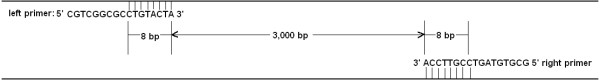
**The definition of minimum amplicon size (MAS)**. MAS is the minimum distance (default = 3,000 bp) between the left and right primers when the left and right primer of a pair have consecutive matches of at least eight nucleotides (default value of MPM) with non-target genomic sequences.

**Figure 6:**
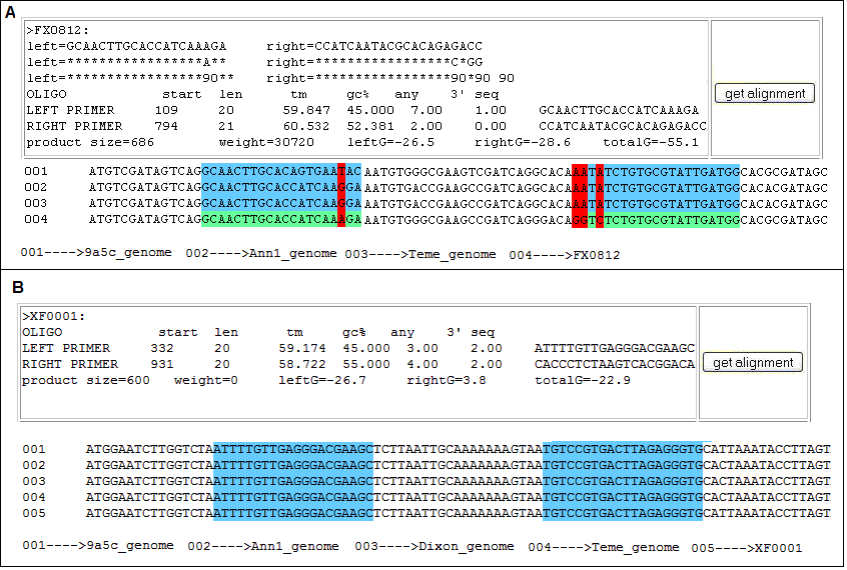
**Sample output of PrimerSNP program**. (A) SPEC output. (B) COMN output. The weight score and the free energy of melting (ΔG°) are displayed in addition to the PCR parameters of Primer3. The sequence alignment file is also available to the user by clicking the 'get alignment' button. The SNP and primer locations are highlighted within the multiple sequence alignment file. Red color highlights SNP locations; green color highlights the specific primer locations and blue color highlights the homologous regions of other stains/species.

The SPEC subprogram consists of the following steps:

1. Extract homologous regions of each query sequence using BLAST search against all the reference genomic sequences. Multiple sequence alignment of these sequences is made with ClustalW2.

2. Identify unique SNPs and generate candidate primers. Based on the multiple sequence alignment file, SNPs that are unique to the query sequence are next identified. A SNP is defined as unique if it is specific only to the query sequence (Figure 2). Using each SNP as the 3' end base, a series of sequences to the left and right of the SNP are selected as either candidate left or right primers (Figure 3).

3. All the candidate primers generated in the previous step are evaluated using Primer3 based on the user-defined PCR parameters. If a candidate primer is appropriate, the second half primer is generated by Primer3, if possible.

4. Calculate the weight score and free energy of melting (ΔG°) of primer-DNA duplex. The weight score is a measurement of the number and the locations of SNPs in the designed primer pair. A weight score of a specific primer pair is calculated for the first 15 nucleotides as shown in Figure 4A. A score of 2^(15 – n) ^is assigned to a base if it is a SNP and its position is n (starting from 3' to 5' end). The weight score of a primer pair is the sum of all the score values of SNPs on the primer sequences. In addition to weight score, the free energy of melting (ΔG°) is also calculated. This thermodynamic parameter is often used to estimate nucleic acid stability [10]. To calculate ΔG° of the primer-DNA duplex, we used the improved thermodynamic nearest-neighbor model [11].

5. Checking the primer specificity. A primer pair is defined as specific if it satisfies the following conditions: a) The primer pair has a perfect match with the target genome sequence only once; b) both left and the right primer do not show consecutive nucleotide matches of more than eight (default value of Maximum Perfect Match (MPM), can be user-defined) base pairs (starting from 3' to the 5' end) with the reference genomes simultaneously; and c) in case of the second condition is not satisfied, the amplicon size should be greater than 3,000 bp (default value of Minimum Amplicon Size (MAS), Figure 5).

6. Sort output primers. The primer pairs are sorted in the descending order of the weight score. The program output format is similar to Primer3 with the additional parameters of weight score and ΔG°. A graphic view of the locations of the primers and their SNPs within the multiple sequence alignment file is also provided (Figure 6).

The COMN subprogram consists of the following steps:

1. Extract homologous sequences for each query sequence.

2. Generate primers for each query sequence using Primer3 according to the user defined PCR parameters.

3. Select those primer pairs that have perfect match with all the other homologous sequences.

4. Calculate the weight score, which is the difference between the amplicon size of the possible PCR product and the optimum product size (Figure 4B). For each query sequence, the primer pair whose amplicon size is closest to the optimum product size is selected.

### Using PrimerSNP

#### Required input

##### Genomic sequences

The genomic sequences of microorganisms (multiple strains or closely related species) are uploaded by the user in FASTA format.

#### Optional inputs

##### Query sequence file

The user can optionally provide a file containing multiple query sequences (in FASTA format) of the target strain for which allele-specific primers are to be designed. In case the user does not provide such a file, the program automatically generate a query file by splitting the target genomic sequences into 1,000 bp fragments.

##### Maximum Perfect Match (MPM)

the maximum number (default = 8) of base pairs (starting from 3' end) of both left and right primer that have consecutive matches with a non-target genomic sequence.

##### Minimum Amplicon Size (MAS)

the minimum distance (default = 3,000) between left and right primers on a non-target genomic sequence (Figure 5).

##### PCR parameters

primer length (bp) (default: 18 to 22, optimum = 20), annealing temperature (°C) (default: 57 to 63, optimum = 60), amplicon size (bp) (default: 200 to 1,000, optimum = 600), GC percentage (%) (default: 20 to 80, optimum = 50).

##### Email address

upon completion of the program, the user is notified by email with a web link to the result.

#### Output result

The output primers are presented in a table format. For each primer pair, the SNPs, weight score and the free energy of melting (ΔG°) are displayed in addition to the PCR parameters of Primer3. A hyperlink button is also provided by PrimerSNP to graphically view the locations of the primers and their SNPs within the multiple sequence alignment file (Figure 6).

## Results

### Common primer design

Forty pairs of primers were designed for 40 homologous genes among the four strains (Temecula-1, 9a5c, Dixon and Ann1) of *Xylella fastidiosa*. The experimental validation result showed that all the 40 common primers successfully amplified the corresponding homologous sequences on each of the strains (data not shown).

### Specific primer design

To design strain-specific primers, 2,766 genes of strain 9a5c, 2,622 genes of strain Dixon, 2,034 genes of strain Temecula-1 and 2,815 genes of strain Ann-1 were used as the input query sequences for specific primer design. When one strain was used as the target, the genome sequences of the other three were used as the reference. These gene and genomic sequences were downloaded from . Except for PCR product length which is set at 100 – 400 bp for Dixon and 200 – 300 bp for others, the rest of the parameters are the default values of this program. PrimerSNP generated 288 specific primers for strain 9a5c, 46 for strain Temecula-1, 136 for strain Ann-1 and 6 for Dixon. Since the number of specific primers for strain Dixon was too low, we tried to increase the number of specific primers by manipulating the input files in various ways. First, Dixon genome sequence was cut at 300 bp interval and PrimerSNP designed specific primers for each individual piece. Next, a similar process was repeated for the same genome sequence that was cut at 500 bp, 800 bp, 1,000 bp and 2,000 bp intervals. Finally, all the specific primers were combined together and the total number was 21. Since the number of specific primers for strain 9a5c was too many, we ranked the 288 primers in the descending order of weight scores. A total of 41 specific primers were selected at an even interval (see Additional file 1). We tested the specific primers with three (9a5c, Temecula-1 and Dixon) of the four strains of *X. fastidiosa *due to the availability of genomic DNA.

**Figure 7:**
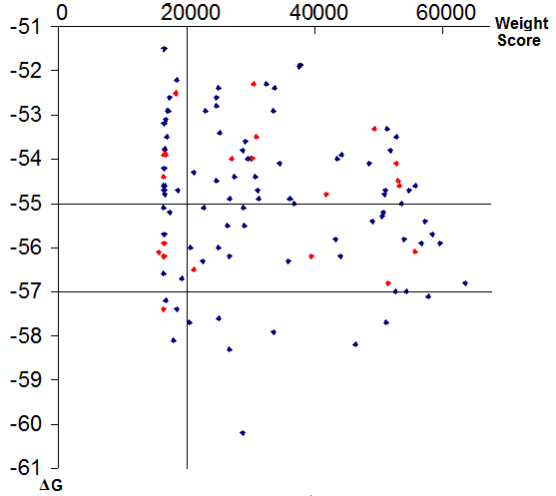
**Validation of specific primers generated by PrimerSNP**. A total of 107 specific primers (amplicon ranges from 200 to 300 bp) of 9a5c, Temecula-1 and Dixon strains were tested using standard PCR. Each pair of the specific primer is displayed according to its weight score (*x*-axis) and the free energy of melting (ΔG°) (*y*-axis). The red dots represent those primers that are not specific, and blue dots represent specific primers.

PCR reactions were done followed by running agarose gels stained with ethidium bromide (EB) after electrophoresis. The results (Figure 7) showed that 80.4% of the strain specific primers were successfully predicted (see Additional file 2). They only amplified the target genomic DNA, while no amplifications were detected using non-target DNA. Further analysis of the result found that the successful prediction rate was influenced by both the weight score and the free energy (ΔG°). If the weight score < 20,000, 75% of the primers were specific. At this weight score level, ΔG° did not affect the prediction rate. However, if the weight score was above 20,000, ΔG° did influence the prediction rate. If ΔG° < = -55.0, 89.6% of the primers were specific; If ΔG° < = -57.0, 100% of the primers were specific. To test the effect of the weight score and ΔG° on the prediction rate, we categorized the data into two groups. Group I consisted of the primers with weight scores > = 20,000 and ΔG° < = -55.0, and Group II consisted of the rest. In both groups, the primer pairs with false positive result were given a value of 0, and 1 otherwise. A *t*-test was done with R-2.6.2 on two independent sample inference with unequal variances showed that the value of Group I was significantly higher than Group II at 95% confidence level with a p-value less than 0.05 (see Additional file 3).

### System performance

The following example files were used to test the system performance: four genomic sequences (totally 13.2 M) of *Xylella fastidiosa *and a file (2.3 M) of 2,766 query sequences. When no other jobs submitted, it took 3.23 hours to complete the design of specific primers and 3.43 hours for the common primers. The program efficiency of Windows version was also tested on a PC with Windows XP (2.8 GHz and 2 G RAM). It took 7.27 and 5.5 hours for specific and common primer design, separately. Although the PC version takes double the time of an online version, it recommends that the user use the PC version since the time for the completion of a job by the server is significantly delayed by the number of jobs in the server.

## Discussion

An advantage of using PrimerSNP is that the generated specific primers are not only allele-specific, but also genome-specific. To ensure genome-specificity, PrimerSNP takes into consideration the partial matches of primers with non-target genomic sequences. The partial sequences of a primer pair, especially sequences close to the 3' end, are critical to primer specificity [2,12]. They may have multiple matches with the target and reference genomes. To eliminate this possibility, PrimerSNP checks the first eight (default value, can be user-defined) nucleotides (starting from 3' to 5' end) to ensure that only one perfect match exists between the partial 3' end sequence and the target genomic sequences, and no match with the non-target genomic sequences.

PrimerSNP uses a binary exponent system to accurately quantify the number and position of SNPs in each primer. The weight score takes into consideration both the number and the locations of SNPs. Thus, weight score is a means to compare the specificity among the primer pairs. No other programs have attempted to quantify the specificity of primers. Our initial thought was that higher weight score could result in more specific primers. However, our experiment showed that both the weight score and the free energy of melting (ΔG°) influenced the specificity of primers. A weight score of 20,000 is critical for specificity. Below this value, 25% of primers are non-specific. If the weight score is greater than 20,000, the lower the ΔG° value, the more specific of the primers. When ΔG° value is less than -55.0, 89.9% primers are specific; when ΔG° value is less than -57.0, all primers are specific. Based on this result, we suggest the following selection criteria for the user: weight score > = 20,000 and ΔG° < = -55.0.

Since the purpose of PrimerSNP is to select reliable specific primers for detection purpose, it uses strict algorithm to filter out those primers that have partial matches with the non-target genomic sequences. Therefore, no specific primers are generated for most query sequences. Only 1% to 10% of query sequences have specific primers designed. The strictness of this program ensures a higher prediction rate (>80%).

PrimerSNP is ideal for bacterial species. It may not be suitable for virus species whose genomic sequences have high mutation rate and therefore many strains. The detection of a particular strain is usually out of the research interest.

PrimerSNP finds its potential usage in screening for risky species and disease epidemics study. The Gram-negative, xylem-colonizing bacterium *Xylella fastidiosa *(Xf) is the causative agent of several important diseases including Pierce's disease (PD) of grape vines, almond leaf scorch disease (ALSD) and citrus variegated chlorosis (CVC) [13]. Citrus variegated chlorois (CVC), caused by the 9a5c strain, is not known to occur in America. Should Xf-CVC be introduced into California, preventing its spread and establishment in citrus growing areas would be very difficult and perhaps impossible. The availability of multiple specific primers could be used to reliably identify this pathogen from thousands of environmental samples. Similarly, specific primer design could be used in identifying pathogens of citrus canker disease, caused by *Xanthomonas axonopodis *pv. citri (Xac), whose genomic sequences are available and no occurrence has been reported in California.

Strain differentiation is another area that PrimerSNP could be used. Since each primer contains unique SNPs for the strain, the change in these SNPs could be easily detected by PCR. Instead of sequencing many target regions, standard PCRs on these strains using specific primers that are designed by PrimerSNP can provide a rapid clue to this question for biologists. Multiple specific primers can also be used for strain genotyping research.

Currently, the specific primer design using PrimerSNP for higher organisms such as fungi is greatly limited due to the availability of genomic sequences. With the rapid development of sequencing technology such as 454, the cost and time would be significantly reduced. PrimerSNP will certainly be a useful tool for higher organisms.

## Conclusion

PrimerSNP is a convenient tool that automates the process of selecting specific primers that contain strain-specific SNPs at the genome level which can be used to reliably differentiate one strain from other phylogenetically-related strain/species.

## Availability and requirements

Project name: PrimerSNP

Project home page: 

(PC version can be downloaded from this main page.)

Operating system(s): Platform independent

Programming language: Perl

Other requirements: None

License: PrimerSNP is free to academic users while commercial users must acquire a license.

## Authors' contributions

EC conceived of the study, JY did all programming, JY and DH and wrote the main manuscript. LH, DA, FM and LE performed the lab experiments, made suggestions to program functionality and edited the manuscript. All authors have read and approved the final manuscript.

## Supplementary Material

Additional file 1 **The specific primers designed for *9a5c*, *Temecula1 *and *Dixon *Strains of *Xylella fastidiosa***. The specific primers designed for strains of *Xylella fastidiosa *using PrimerSNP. Click here for file

Additional file 2 **The result of experimental validation of the specific primers designed for *9a5c *, *Temecula1 *and *Dixon *Strains of *Xylella fastidiosa***. The data provided the experimental validation of the specific primers that are designed using PrimerSNP. Click here for file

Additional file 3 ***t*-test on the effect of weight score and free energy (ΔG°) on the specificity of primers**. The data provided the statistical analysis of the primers' specificity of two groups' primers that have different values of weight score and free energy (ΔG°). Click here for file
