# Supplementary material for: PrimerSNP: a web tool for whole-genome selection of allele-specific and common primers of phylogenetically-related bacterial genomic sequences
Source: BMC Microbiol. 2008 Oct 20;8:185. doi: 10.1186/1471-2180-8-185 (PMC2579435; doi:10.1186/1471-2180-8-185)
Supplement: Additional file 1 — The specific primers designed for 9a5c, Temecula1 and Dixon Strains of Xylella fastidiosa. The specific primers designed for strains of Xylella fastidiosa using PrimerSNP. [file 1471-2180-8-185-S1.doc]

Supplementary Table 1 The Specific Primers Designed for *9a5c*, *Temecula1* and *Dixon* Strains of *Xylella* *fastidiosa*

| No. | Strain | Gene | Weight | ΔG° (kcal/mol) | | | Forward Primer | Reverse Primer |
| --- | --- | --- | --- | --- | --- | --- | --- | --- |
|  |  |  |  | Left | Right | Total |  |  |
| 1 | 9a5c | XF2039 | 63462 | -27.5 | -29.3 | -56.8 | CGATTTCTCACGTCCTCACT | ACGCAAAGCTCGCTGTCTAC |
| 2 | 9a5c | XF1774 | 59574 | -28.1 | -27.8 | -55.9 | TGCCTATGTGGTCGGGTAAG | GAACCGCTGACGAAAGAAAG |
| 3 | 9a5c | XF0328 | 57709 | -28.7 | -28.4 | -57.1 | GGTTGCCGCTACTCCACTAC | ATGTCGCATCGGCAGTAGAA |
| 4 | 9a5c | XF1761 | 57209 | -27.7 | -27.7 | -55.4 | CTTCGACGTCAAGGTCTCG | CTGCTTGATCGAAGCCAGTT |
| 5 | 9a5c | XF1719 | 56679 | -28.9 | -27 | -55.9 | CACTCAAGACGCAACCTTTGT | ACACCTGCTGGATGTTCTCT |
| 6 | 9a5c | XF2741 | 55710 | -27.1 | -27.5 | -54.6 | TGGCGAATGGCATAGAAAGT | TGTGGCAGAACAACTGGGTA |
| 7 | 9a5c | XF1781 | 55644 | -28.5 | -27.6 | -56.1 | CCTCGGCAACCTCTATGTCC | CCTTCTTCGGAATCGTGAGT |
| 8 | 9a5c | XF2294 | 54723 | -27.7 | -27 | -54.7 | TCGTGTGTTTCTTGCCTCTG | CTGCGGCCTCTTTAAGTCC |
| 9 | 9a5c | XF1770 | 53960 | -27.5 | -28.3 | -55.8 | TGAACCTCACGGCTCAGATA | ATGCCCGATGACGAAGAGTT |
| 10 | 9a5c | XF2745 | 53241 | -26.8 | -27.8 | -54.6 | TGACTGACTGCCGGGACA | AGCGTGCATCAATACTCTCG |
| 11 | 9a5c | XF1733 | 52946 | -28.1 | -26.4 | -54.5 | CTACGGCCATACCGAAAGAC | GTTGCGCCACTTCTGCTC |
| 12 | 9a5c | XF2584 | 52780 | -26.0 | -27.5 | -53.5 | CTGCGCGCTTTTTGTTCT | CACCTGCTGATCTTGCTAAAC |
| 13 | 9a5c | XF1755 | 52687 | -27.2 | -26.9 | -54.1 | GATGGAGTCGGTGTTTCACA | GTTGCGCGACGGATAGAG |
| 14 | 9a5c | XF0689 | 52547 | -29.0 | -28.0 | -57.0 | GTCTTTTGTGCCGACAGAGTC | GTTTCGCTGTGTTGTTCACTT |
| 15 | 9a5c | XF0779 | 51805 | -27.6 | -26.2 | -53.8 | TCGAAGAACTCGGAATGGAG | TGATTCGATAACCCCCTTTG |
| 16 | 9a5c | XF1384 | 51408 | -27.7 | -29.1 | -56.8 | ACGTCGTGCTTTACATACCC | ATGTGCGACACTCTCGTCCT |
| 17 | 9a5c | XF0696 | 51270 | -26.2 | -27.1 | -53.3 | TGCTCAATGGCTCGAAACT | TACCCGTGACGAGCACTAA |
| 18 | 9a5c | XF0897 | 51107 | -28.8 | -28.9 | -57.7 | GAAGGCAACCGTCTTACAGAG | GCGGTGAGGTTCAGAGTGTG |
| 19 | 9a5c | XF2037 | 51069 | -26.0 | -28.7 | -54.7 | CTTCCTGGACCATTGAGAAG | GATCTCGACCGAGAGCTGAG |
| 20 | 9a5c | XF2715 | 50861 | -26.8 | -28.0 | -54.8 | TCTCCACCAAGGATGAGCTA | CACTCCGCAGAAAGTTCGAT |
| 21 | 9a5c | XF1743 | 50683 | -27.6 | -27.6 | -55.2 | AGGATCGTCTCGGTCAACTA | CGTTGGTGAAATAGCTGTCG |
| 22 | 9a5c | XF1727 | 44085 | -28.6 | -27.6 | -56.2 | GACCGACATCGTGGACTTCA | TCCATGAACCCGTAGTTCCA |
| 23 | 9a5c | XF0496 | 39350 | -27.2 | -29.0 | -56.2 | GGTAGCATGGCCGAGATAAA | ACGCGGAATAGCAGCAAAGT |
| 24 | 9a5c | XF1772 | 36815 | -26.9 | -28.1 | -55.0 | CATCCCGTGAGGGACGAG | CGGCATAGCTGCGGAAGTA |
| 25 | 9a5c | XF0491 | 33424 | -27.3 | -25.6 | -52.9 | TATCGGTTGCGCCTATGAAT | GCCTTTGGCGTAGCAGTA |
| 26 | 9a5c | XF0935 | 32486 | -26.6 | -25.7 | -52.3 | CGTGGTGAATTTCTTTCGAG | CAATTAGCCCAACGTCCAA |
| 27 | 9a5c | XF1008 | 30497 | -25.6 | -26.7 | -52.3 | AATTGGATGCAGGGAGAGG | GATGGTGAATGCGACGATTA |
| 28 | 9a5c | XF1437 | 28676 | -27.2 | -26.6 | -53.8 | CAACAGTTTCAGCCTGTGGT | GCTCCCCCTTCAATGTCTTA |
| 29 | 9a5c | XF0693 | 26624 | -28.9 | -27.3 | -56.2 | AAAGCGCCGTTGTTTTACTTC | ATACAGTGGATGCGTTGTGG |
| 30 | 9a5c | XF1358 | 24864 | -28.2 | -27.8 | -56.0 | TTGGTTTGAGGGCGGTCTTA | TCCAACCACACGCCCTAGT |
| 31 | 9a5c | XF1815 | 24608 | -26.2 | -26.4 | -52.6 | TGATGAAGAGCTGCGAGTC | CCATTTGGTTTCCACTGCTT |
| 32 | 9a5c | XF0323 | 22593 | -27.4 | -28.9 | -56.3 | ATGATCAGCATCGCTCTCCT | TCGCCATACCCAGTTATCGTA |
| 33 | 9a5c | XF2091 | 20616 | -27.5 | -28.5 | -56.0 | GCTGTGGGAATGACTTTCGT | CGCTGTGGTTGATTGTCCATA |
| 34 | 9a5c | XF1647 | 19267 | -29.1 | -27.6 | -56.7 | CAGCAGTAACGCCACATTAGG | ATCTGGGAACAGGGAGAGGT |
| 35 | 9a5c | XF2759 | 18560 | -26.7 | -28.0 | -54.7 | GGATCGGATCGAAGAGAGC | CGTTGTAGCCAACGAGGTAA |
| 36 | 9a5c | XF0486 | 18392 | -25.8 | -26.4 | -52.2 | ATTGGCGCAGGATCATACA | CCTGATAAACGCCCTATCAC |
| 37 | 9a5c | XF1785 | 17226 | -25.5 | -27.1 | -52.6 | CCATCATTTCAACGAAAGGT | GAAATGATTGTGCGGGACAC |
| 38 | 9a5c | XF0883 | 16649 | -27.2 | -26.6 | -53.8 | GATGCCCGTGGCTCTTACT | CTAGCGCGTGGTCAAGTG |
| 39 | 9a5c | XF0321 | 16512 | -28.0 | -27.9 | -55.9 | CAACACTGGACGGTTCACAC | ATGCCCGGGTATGGTCTAAG |
| 40 | 9a5c | XF2426 | 16420 | -28.2 | -28.0 | -56.2 | CGGTACGCTGACTCGGTTA | GCGTCGACCGGATAAGTATT |
| 41 | 9a5c | XF0595 | 16393 | -27.1 | -27.5 | -54.6 | CACCACACCAAACGGTAGC | GGGTGTTTTTGGGTACTTCTG |
| 1 | Temecula1 | PD1608 | 54247 | -28.7 | -28.3 | -57.0 | CACGCGAAAGGCTACGAGT | GCGGTCACTTCTGATTCGAC |
| 2 | Temecula1 | PD2097 | 50439 | -27.4 | -27.9 | -55.3 | CAGGCTCTGGTTATGGTCCT | ATTCAGGTGCGCGTATAAGG |
| 3 | Temecula1 | PD2071 | 49017 | -27.6 | -27.8 | -55.4 | ATAGTTATCGGCCGGAAAGG | GTTCAGCGCATCGGTTCTG |
| 4 | Temecula1 | PD0304 | 48542 | -25.0 | -29.1 | -54.1 | GGGGCCACTGGAAATCAA | CGGGGTAACGCCTTAAGATAG |
| 5 | Temecula1 | PD2075 | 46371 | -27.8 | -30.4 | -58.2 | ACGCTACTGCCTCATTCCTT | AACTTCTACCGGCAGTCTAGGA |
| 6 | Temecula1 | PD0833 | 44121 | -26.9 | -27.0 | -53.9 | GAGACGCAGGATAAGGCATA | GAGCCGTGGCGTGTTATTA |
| 7 | Temecula1 | PD1507 | 43520 | -27.3 | -26.7 | -54.0 | TGGTAGTGCCGAGGGATATT | CCCAATGCCACCTCATAAGT |
| 8 | Temecula1 | PD1607 | 43235 | -27.6 | -28.2 | -55.8 | TTTTGGTCACGCAACGTAAG | CTTGTAAAGGCCGCAAATCG |
| 9 | Temecula1 | PD0088 | 36128 | -27.9 | -27.0 | -54.9 | TCTGTGAACACCTGCGCTAT | GACGAATGCGGCTACACTT |
| 10 | Temecula1 | PD0087 | 35906 | -28.4 | -27.9 | -56.3 | GCTTCAGCATCGCTTACGTT | GATGCCATCACTGGTTAGGAC |
| 11 | Temecula1 | PD1014 | 33797 | -25.8 | -26.6 | -52.4 | CGTGATGGAAGCATGTGAG | GCTGCTGGTGGTTGAACTC |
| 12 | Temecula1 | PD0920 | 31110 | -28.2 | -26.5 | -54.7 | GGGGGCGTTATGTCAGAATTA | TGTTCCACACGCTTAACAAA |
| 13 | Temecula1 | PD1190 | 30783 | -26.8 | -26.7 | -53.5 | ACGATCAACCGGCCTGAC | CCGATGGCGTCTGTGACT |
| 14 | Temecula1 | PD2094 | 30097 | -28.6 | -25.4 | -54.0 | CAGGTGGTGCCAGTGTTAGC | GATTCGCGTCGCATCTTA |
| 15 | Temecula1 | PD1283 | 28932 | -28.2 | -27.3 | -55.5 | CTCCGGCCTGGTATTGAACT | TTGAGGTCCCTCCCTTAGTG |
| 16 | Temecula1 | PD1242 | 28868 | -27.4 | -27.7 | -55.1 | TGGCAGGCGTAGTCAGAAG | TAGCTGCAAAAAGACCTTCCT |
| 17 | Temecula1 | PD0305 | 27440 | -27.9 | -26.5 | -54.4 | GATGCGGGACGTTATGACAA | TGGTCCAATCCTGGACAATC |
| 18 | Temecula1 | PD1349 | 27065 | -26.5 | -27.5 | -54.0 | CCAGCAGCCAGAGCAGAG | CGTTGCGTGAGTACGGAAT |
| 19 | Temecula1 | PD1951 | 26645 | -30.0 | -28.3 | -58.3 | CGCCATCGCTAGTCTAGTCAC | TTGGCGAGCGAACCTAAAAA |
| 20 | Temecula1 | PD1506 | 26294 | -28.0 | -27.5 | -55.5 | GCATCAGGCCGATCTGTACT | CGCCAAACATGAGAGCATTG |
| 21 | Temecula1 | PD2109 | 25160 | -25.0 | -28.4 | -53.4 | ATGCATGGAATTCCTCCTAA | AGTCGTCTGCAAAGAACCAAA |
| 22 | Temecula1 | PD1434 | 25034 | -27.9 | -29.7 | -57.6 | GCGATGGACTGTAGCAAGTG | TCCGCAATAGGCGTTACAACT |
| 23 | Temecula1 | PD1924 | 24644 | -28.4 | -26.1 | -54.5 | GTAAGGTGCAAGCCGAGTCT | TGGTGTTGCATGTCCACTG |
| 24 | Temecula1 | PD1002 | 24644 | -27.7 | -25.1 | -52.8 | GGCAACTCAGAAAAACCTGTC | GCTTGCGGAATCTTGTTTT |
| 25 | Temecula1 | PD1416 | 21058 | -28.3 | -26.0 | -54.3 | CGCTGACCTTGCTTATGGTAT | CATGTTTGGTGGAAGTTTGC |
| 26 | Temecula1 | PD2108 | 21025 | -27.6 | -28.9 | -56.5 | GATCGCAGGGATAGAGGTCA | CCATCTTGCTTGAGTCACGTC |
| 27 | Temecula1 | PD1362 | 20481 | -27.9 | -29.8 | -57.7 | GCTTAGCCTTTGGTCTCTCG | ACCTCCTGCACTTGCACTTGT |
| 28 | Temecula1 | PD0502 | 17440 | -26.8 | -28.4 | -55.2 | ACATGCGTCCTGATCTTGAA | CCCGCTTGGCCTTAGTATCA |
| 29 | Temecula1 | PD1136 | 17132 | -26.5 | -26.4 | -52.9 | AGTCACACCGCCTACGAG | GCTTTGCGCAGGTGAATAC |
| 30 | Temecula1 | PD2030 | 16804 | -27.1 | -26.4 | -53.5 | CTGATGCCGCAGGTCTCTA | TTAAATCCCGTCTCTCATCG |
| 31 | Temecula1 | PD0996 | 16768 | -27.4 | -26.5 | -53.9 | CGGTACGGCGACATGTGA | TTTTCGACGCCGAACTAAA |
| 32 | Temecula1 | PD1323 | 16768 | -27.4 | -25.7 | -53.1 | CGGTACGGCGACATGTGA | GGAATTGGCCTTGGGGTAT |
| 33 | Temecula1 | PD0980 | 16708 | -29.5 | -27.7 | -57.2 | GCTGAGGGAGAACCTTACGAC | CTCTTCCAGGGCATCCACTT |
| 34 | Temecula1 | PD1107 | 16640 | -27.1 | -27.7 | -54.8 | CATCAATGGACGAGGCTACA | CCCTCTGGGTAGCAGACAAA |
| 35 | Temecula1 | PD1125 | 16514 | -27.2 | -27.5 | -54.7 | CATCGTTAAGCACCCAGACT | CTTCAACCACTGAGCCGTAA |
| 36 | Temecula1 | PD0579 | 16456 | -28.1 | -26.1 | -54.2 | CGTAAGCAATTTGAGGAGGTG | ATTGGCACCGTCTGAACG |
| 37 | Temecula1 | PD2080 | 16448 | -26.0 | -27.2 | -53.2 | GACAGTGCCCAAACTGTCA | AGCATCAGGCTCACCTTGTA |
| 38 | Temecula1 | PD1240 | 16420 | -26.4 | -27.5 | -53.9 | TGGTGATTGCAGGGATAGAG | CTCAAAGTCGGGTTCTTTTCT |
| 39 | Temecula1 | PD0993 | 16416 | -28.5 | -27.2 | -55.7 | TGGCAGAGACGGGAAGAAAG | GCCAAAAGATGATGGCTGTAA |
| 40 | Temecula1 | PD1223 | 16416 | -24.7 | -26.8 | -51.5 | CTTGATGAAGGCGCAAGT | GCTTGGGTTTGTATGGGAGA |
| 41 | Temecula1 | PD0478 | 16400 | -29.5 | -27.1 | -56.6 | ACCCCGCAGATGTACGAGAG | TAAGTGAGCGGCATCCAAAT |
| 42 | Temecula1 | PD0960 | 16393 | -27.2 | -27.9 | -55.1 | TTGACCGGCTACATTCTCAC | CGCTCATGAAAAATAGCCACA |
| 43 | Temecula1 | PD1451 | 16392 | -29.3 | -28.1 | -57.4 | GCGGAGATGGTTTGTCAGAGT | CCCCCATGTCGAGTGTGTAA |
| 44 | Temecula1 | PD0817 | 16384 | -26.3 | -29.9 | -56.2 | TAGGCATCACCCGGATACA | ACGGTCTCTCCATCGACAGTT |
| 1 | Dixon | FX0.3K05203 | 16400 | -27.0 | -27.4 | -54.4 | TGGCAATACAGCGAGTCATT | GCCACGAAAAGTCCCTAAGA |
| 2 | Dixon | FX0.5K03011 | 16672 | -26.9 | -27.7 | -54.6 | GCCACTGCTGCGACACTT | GCAGTCATCACACTCCGTTC |
| 3 | Dixon | FX0.5K03514 | 16964 | -26.2 | -26.7 | -52.9 | TCGGCAACTCGTTATACCA | GCTTTGGCTTGGGTTATCAG |
| 4 | Dixon | FX0.8K03268 | 17905 | -28.1 | -30.0 | -58.1 | AAGGCAACGGTAGCAGAATTA | AGTGTCGCGAGGTAGGTACG |
| 5 | Dixon | FX0.5K03872 | 18289 | -25.1 | -27.4 | -52.5 | TAAAATCGGCCTGGAACG | AACGTCCCGATTCCTATTCC |
| 6 | Dixon | FX0.5K05247 | 18505 | -28.8 | -28.6 | -57.4 | ACGTGTCGACGGAACTTGTA | GAGCCTGGTCGACTCCTAGA |
| 7 | Dixon | FX0.8K01884 | 22608 | -27.8 | -27.3 | -55.1 | CGCTGTTTGACAGGAAGTTTA | GAGCGCGATTTACCAACAAT |
| 8 | Dixon | FX2K00754 | 22912 | -25.8 | -27.1 | -52.9 | GCTGGGCATCAGGTTTAATA | AGTGAAATATGCGGGCACTT |
| 9 | Dixon | FX0.5K05114 | 24914 | -25.6 | -26.8 | -52.4 | CCTTCTGGGGTACGAAGC | ACCGCCACAAAATCTATTGC |
| 10 | Dixon | FX1K02614 | 26787 | -27.1 | -27.8 | -54.9 | GCCTGGTGGTGGTAAGAATC | CGGACTATCGAAAAGTTAGCA |
| 11 | Dixon | FX0.5K05228 | 28686 | -30.5 | -29.7 | -60.2 | TAGTCCGGAAAGTTCTCCCTGA | CGTAGACGTCGCCTAAAACAA |
| 12 | Dixon | FX0.5K05115 | 29148 | -26.5 | -27.1 | -53.6 | GGTGCGACGGTTGATTCC | AGCAGGTTGCTCCCTCCTA |
| 13 | Dixon | FX0.8K00387 | 29559 | -27.0 | -27.0 | -54.0 | GCGTTCAGAGCCACGACT | TGCCTGCAATGATCTCGTTA |
| 14 | Dixon | FX0.8K03197 | 30693 | -28.3 | -26.1 | -54.4 | CTTCGCGCTCTTTTCTGACT | GGGCAACAATCTACAGGATG |
| 15 | Dixon | FX0.3K08525 | 31284 | -27.8 | -27.1 | -54.9 | TTGGCTGGGAGAACTAAATCC | AGCAGGTTGCTCCCTCCTA |
| 16 | Dixon | FX0.3K08743 | 33558 | -28.4 | -29.5 | -57.9 | CCAGGCTCCAGCTTAACGAT | GGTGCGTGACTCGTCCTCTA |
| 17 | Dixon | FX0.3K08523 | 34468 | -27.3 | -26.8 | -54.1 | TGGAATCACCACAACCAGTTA | GAAACAGGAAAAGGCGAGAA |
| 18 | Dixon | FX0.5K05227 | 37617 | -26.7 | -25.2 | -51.9 | TGATCAACGCGGATATAATTG | CAATGGGAAAAAGTCATTCG |
| 19 | Dixon | FX0.3K05489 | 41715 | -27.4 | -27.4 | -54.8 | GACGCTTCCATCAACTTACG | GAATTGCCGGAACCTCTCTT |
| 20 | Dixon | FX0.5K03294 | 49262 | -27.1 | -26.2 | -53.3 | AGACACCAATCCAGGCCTTA | GCATCGATAACCACGAAGG |
| 21 | Dixon | FX0.3K08524 | 53455 | -28.3 | -26.7 | -55.0 | CTTCGCGCTCTTTTCTGACT | CCACCATCAATCAAGAGCTTA |
| 22 | Dixon | FX0.3K08522 | 58432 | -28.1 | -27.6 | -55.7 | ATAAAACCCTCGCTAGGGAAC | GGTGAAGTCGTGCTCGTTG |

Note:

Note: 1) The total free energy (ΔG°(sum) = ΔG° (forward primer) + ΔG° (reverse primer)) value was used in this manuscript.

2) All the gene sequences of 9a5c and Temecula1 strain and their upstream and downstream sequences are listed in <http://cropdisease.ars.usda.gov/CVC_index.htm> For the DNA sequences of Dixon strain, they are shown below.

The DNA Sequences of the 22 Fragments of Dixon Strain

| No. | Name | Sequences |
| --- | --- | --- |
| 1 | FX0.3K05203 | GTTTCGGCATTATTGGGCCTATTACGTGGCTTTGTCAGCATTATAATAAGTACCTCCTCTTGGCTACTGGCCAGCTGGGCGACATTTGAGTTTGGCAATACAGCGAGTCATTGGCTAGCAAGCCATGGTATTCCATCGACTACTGAAATATTGTGTGGTTATGCGTTGGTGTTCGTCGGGACTTTGATGACAGTTGGCGCAGTGGGTATGCTGATGCACGCTGGGATCAACGCAATACGTTTAAACAACATAGACCGCATATTCGGCTTTGTCTTAGGGACTTTTCGTGGCGGCTTCATT |
| 2 | FX0.5K03011 | AACGGAATAATGTACGAATGGCGCAACGGAACAAAACCGATACCAGACGAACGCATCCGACAACTAGCAAAAATTGCTAAAGAAGATGCAGGAAAATGGCTACTACTTATCAGATCAGAACAAGATAAAGGAGAATTAGGCAAAGAATGGGAAAAGCTATACAAAAAACTAACCGCCACTGCTGCGACACTTTTAATAGGCGCAAGCATCACCTGCCCTAACACGTCTCAAGCACAGTCAATAAATAAGAACTTCAGTAATGAAAATTCAGATAATGCATATTATGTCCATTATGTCCATCAGGTGGGCGCTTATAAAGCGCGATGTTTAGCTTCACCAAAGAACGGAGTGTGATGACTGCAATCGTTTAAGCGTACTACTTGTAGTTGGTCAATATTCTTTCCTTCCAAGAGGTTCAGCGTAGTCGGTGCTTGATGGAAGCGCCATAAATGAGCCAATGGCAATCCCAGCACTCGACACAGGATCACTCGGTTGACTGC |
| 3 | FX0.5K03514 | AAATGGTCCTTGTGGAGTGAAGGCAAGGTCTCAATGGCTACTGCATGTTTGCTTAGATCGGCAACTCGTTATACCAGTTGTCCAATATGCTTGGACGTAGTACATGTGGATCGACCTTGAAGCGGCATTACGTGGAGTGTGGTGCAAAAGACGGTGGGCTGTCGTAGCCGCAGTACCATCAGCAGAGCCGTCAAACACGCACAAAATTTCTCCAAGACGTAACTGAAGCAACTGATGATGCGCTGGATTTCAAACGGCCATTATGGGCTACTCGCACGCGAGGCACGCAATGTACAGCTCTGTCTAGTACACGCATTACTAAATACCGCCGATGCACAGCACTACTGTGCATCGGCTTCCATACTTAGATTAGAAACTCAGCTGATAACCCAAGCCAAAGCTATCGCCTCGGCTACGAGCACCAAAGAAGTGTTCTACACCCATGCTCCACTCTCCTGCCGTCATCTCCACGGGCCAGACCATTGATAAGCCAAGAAGTC |
| 4 | FX0.8K03268 | CGTAAGGCAACGGTAGCAGAATTAAGAAATGTCATTGCTTTATTTTCACTTACCGATTGGTAATTTACCAAAGGGTATTGACTTGTAATTATCATATGGTAATTTGAGTGCATGGAAACCCTGCGTACCTACCTCGCGACACTCATCCCAGCCGAACAGGCGCACTACGCTCGTAAAGCGAATACCACCATTGGTTACCTGCGAAAGGCTCTCTGCAAGGGACAGCGATTTGATGGTGCACTGGCGCGACGCCTTGATGAAGCCAGTGGTGGCCGTGTTTCTCGTTACGATCTTCGCCCAGACGTATTCGGCGCTCCCCCCACAGGCCACAGGCAGGAGGTGTCCGATGCGGCGTGAGAAAGGCACTTTTTTTACTTCGCTGTGGGGTGCTTCTTTACGTACCCTTTTATCCATTTGTGGAGGACGATCATCCAAGCCCACCTTGCCACTTTTCTGTAAACGACCGCCACGGTCACAAGTGAAAGCCCGGAAAGACTTATTGCTTGAATTAGATTTGAGTCGAGGATTCGCAGGCAAGCCGATGCGATGGATGTTGGGGAAATCTGAAAAACCCAAAGCAACATCAACGCACACATCCACAAAGCAAGATGAAACATGACGAATATGAAGACTATCGCTAATCGCCCTAAGCGCATGGCCATGTACAAGGCCAACTTCGAGTCGTCTAGCTCCTCTAATTCGCTCATGCCGATCTCCGGTAGTGATTTGGTTGCTTGGAAACACCAAGTCTACCGGCAGATCGGCTCCAACCCGATTCTGATGCGCTGTATTCGATCTTT |
| 5 | FX0.5K03872 | GGCTTTTTTGATGACCTTATACAGACACCTTGGTGGGTATCTGCGATATTGAGCGTACTAAGCGGTACCTTTTTCTTTCTAATAGCGCCTCGTTTTCATCTGCCAGATATTGCAACGAACCCGTTTATGTTAACGCTTGATACGGCGATACACACACCAGAGCTTCTGTATAATATAGGGTGGGTTTTAGTTCTATTTTTCATGACTACAGCCTTGTTCTCATCATGCAGAGAATTGTGGAACTGGTGGAACACACGGTGATTTAACTGCTCCTTATGGCTAAGTCACTACTCGATAAAATCGGCCTGGAACGCTCGAACAAGCTGATGCGTGAAGCCACGCACAAGGCCATCGCCGATGCGCACGCGCACGGCCTGTCGGTCACAGCGGACGTGGGCGGCGTGCTCTCCGAAATCTTTCCTGACGGCCATGTGGAACCCGTGCGGTATTCAGCACATCCGGAATAGGAATCGGGACGTTCAGTATGTCTTAATTGAATA |
| 6 | FX0.5K05247 | GCATGCACCGCAGTGGTGCAATACACGTCGACTGCAGGCATGCATGGCATGCTCGGGTCACTGGAGGATCCTCTAGAGTCGACCAGGCTCCAGCTTAACGATATTTGGAGGTCAGCACGGTGCTCACGTGTCGACGGAACTTGTACAGGGAGGCATGCACCGCAGTGGTGAATACACGTCGACCAGGCTCCAGCTTAACGATATTTGGAGGTCAGCACGGTGCTCACGTGTCGACGGAACTTGTCCAGGGAGGCATGCACCGCAGTGGTGAATACACGTCGACCGCAGCATGCATGCATGCTCGGGTACTGGAGGATCCTTCTAGGAGTCGACCAGGCTCCAGCTTAACGATATTTGGAGGTCAGCACGGTGCTCACGTGTCGACGGAACTTGTACAGGGAGGCATGCACCGCAGTGGTGAATACACGTCGACCAGGCTCCAGCTTAACGATATTTGGAGGTCAGCACGGTGCTCACGTGTCGACGGAACTTGTCCAGGG |
| 7 | FX0.8K01884 | ACTCGACGCCATCTTTGGGTGAATAGCCTTTCACGTGACCGAAAGTAATACGTAATGATAAGTTGGCGTCTGGGTAAACAAATTTTCCATGGCTCTTGTTGTAATCAGCTAAGGCCTGCAGGTAGATCGGGCGTGCTTTGAGTAGTTCTCCAGTACGGATCTTATTTTGACGTTCAATCTCCAGTAATGCTGGCATGATGGCCACCGCATAACGGATCGCCGGATCTTGACTGCTTTCAAACACCGCGCGGTCGGCCTTGAACCACTTCAGCCTTTCCTCAGAACTGCTTAATTTGGTGTCATCCAAGCGCTTGAGGGTTCCCGTGATGCCATCTCCCAACCAAGCATCAATTGCAGCCACACGCTGTTTGACAGGAAGTTTAGTGTATTCGGTGAGCCAATACTGCTGCATCTGGCGGTCCATTGCCGGTACGTAGCGCCGCTCCATCTGCTTTAAGTTACCCTCAATTGTTGGTAAATCGCGCTCCTGATATCCCGCTTCACGCTGGGCATCAGGTTTAATACGCTCGATTGCTAAGCGATATAATTTCACCGCTACACCAATGACACCGCTTCCGTTGAACTGACCCAGTACAAAATCAAGATCCTGGTTTGCTTTATATTGTTCCGTTAGATCAACCAATGTTTGATACGCAGACAATGCTTCGTGTCCGCGTATGCTCTGCTGTTTCAACCAGGCTAATACTGCCGTTTCCTCACGCTGTTTCTGACCCATAGCATCAATGCGTCGGAAGCCTTCCAATTGACCATCAAAGTTTTTCGATACGTTACTCAATTCA |
| 8 | FX2K00754 | ATGACGGCGTTTGCAGCAGTTTAACGATACAAATTCAATTCCTTTAGTAGACGTGGTGCTGGTGCAACTTCAGTCATGATCCACTGCACATAACGACTATCAACTGCAATTGTTCGAGTCATCACAGGATCAAATACCCAATTACTACTGACAGACTCCCAATTACCGTCAAATACTAAACCTACCAATTTTCCGTGGGCATCTAAGACTGGCGATCCGGAGTTGCCGCCGGTCATCTCCAAGTCAGATAAAAAATTCACCGGTACAGTGCCAATGCGTTGATCGGCTAAATTGGCATAGTGCTTTGCCTTGATCGCATCAATCAGTGACTTCGGTGAATCGAACGGCTCGACACCTGTATTCTTTGCCATGACACCCTGCAGTGTCGTAAATGGTGTGTACTCGACGCCATCTTTGGGTGAATAGCCTTTCACGTGACCGAAAGTAATACGTAATGATAAGTTGGCGTCTGGGTAAACAAATTTTCCATGGCTCTTGTTGTAATCAGCTAAGGCCTGCAGGTAGATCGGGCGTGCTTTGAGTAGTTCTCCAGTACGGATCTTATTTTGACGTTCAATCTCCAGTAATGCTGGCATGATGGCCACCGCATAACGGATCGCCGGATCTTGACTGCTTTCAAACACCGCGCGGTCGGCCTTGAACCACTTCAGCCTTTCCTCAGAACTGCTTAATTTGGTGTCATCCAAGCGCTTGAGGGTTCCCGTGATGCCATCTCCCAACCAAGCATCAATTGCAGCCACACGCTGTTTGACAGGAAGTTTAGTGTATTCGGTGAGCCAATACTGCTGCATCTGGCGGTCCATTGCCGGTACGTAGCGCCGCTCCATCTGCTTTAAGTTACCCTCAATTGTTGGTAAATCGCGCTCCTGATATCCCGCTTCACGCTGGGCATCAGGTTTAATACGCTCGATTGCTAAGCGATATAATTTCACCGCTACACCAATGACACCGCTTCCGTTGAACTGACCCAGTACAAAATCAAGATCCTGGTTTGCTTTATATTGTTCCGTTAGATCAACCAATGTTTGATACGCAGACAATGCTTCGTGTCCGCGTATGCTCTGCTGTTTCAACCAGGCTAATACTGCCGTTTCCTCACGCTGTTTCTGACCCATAGCATCAATGCGTCGGAAGCCTTCCAATTGACCATCAAAGTTTTTCGATACGTTACTCAATTCAGCTAAAGTGCCCGCATATTTCACTTGAATTTCTGGATTCTGTTTGCCAGCCGCCTCGATCAAAGCAATGAGATTCTTGAAATGCTGGCCAATCACCGGATAAGTCCAGTGAGCAGTATTCTCAAATTCGGCGACCAAAGCATAACGATTGGTCCGTCCTGGGTAACCAGCGACCATCACGAAATCACCATTACCTAATGGTTGATCGCTAAATTTCAGCCAGTGTTTTGGGCGGTAGGGTATGTTTTCTTTAGAGAAGCTCGCTGGTTTACCGTCTTTACCGATGTAAGCACGGTAGAAAGAAAAGTCAGCGGTGTGACGCGGCCACATCCAGTTGTCGACCTCTCCCCCGAACTGACCCACACTCACTGGTGGTGCGTAAACTAAACGCAGGTCCTTGATCTCCAGGTTCTTGGAGACTCTGTAGATATTGCCACCGGCGAAACTGAAGAATTGGCAGTGATAACCCTGTTCGGCCTCACATTTGGCAATTTCTTGTTTGCTGAATGTCTCCAACGCCGTCGTACGCTTGAAGGGGTCATTACCAGCAGCAGCCAGAGCAGCTTTAGCTTGAGCGGTGACATCGGTAATCTGCTCAAGCACGTAAATACGCGCATTCGGACCGGCGCTGACTTCATCAGTCTGAGTCAACGCATTGAACCCTTCTTTGATTAAGTTCTTTTTAGGCGTGGAGTTCAACTGAATCGCACCATAAGCGCAATGATGGTTAGTGATCACAAGTCCTTCTGGTGAAACCAGACTCGCGGTGCAATTGCCGAGAGAGACCACTGCCCCCATGGG |
| 9 | FX0.5K05114 | TTGATCTGCTGGCCATGCCCAATATTAATGAGGTAAATGCCGTCCCCATCAAACGACTGAATACCCGTATCAACGAGCACGACTTCACCAGGCTGGATTACTGGAATCATGGAATCACCACAACCAGTTATTAGTTTCAGCCTTCCCTTCTGGGGTACGAAGCCAATTAAGCTGCGAATGAATGCCTCAGTATATTCCACCGATCGGATGACATCTGGGTAATCTAGATTCACGCGGCTCTCACCCATACCTGCCTCCGCATCTAAATGTTCAACGCGGATATAATGTTGCGATATTTTTTCTGTTGCAATAGATTTTGTGGCGGTTGTGGCTTCGATTTTCTCGCCTTTTCCTGTTTCCAGCCATCGCAAAGAAACGCCCAACGCATCAGCAATAAGTCGAAGCTTTGTAGTCGTTTGCATTCCACCACGTTCCAATTCAGCAATTGTGCTGTAGCCGATCCCTGTTTTCTTGGCGAAATCGGATCTGTTGATGTTCTG |
| 10 | FX1K02614 | ACGAAGTCAGGATAAAGAGCTTTATCCATGCTGGTAATGTATATGTATCCGTTTCTATCATGTAATGCTTTTATTTGTTGTCCGTTACCTGTATTGACTAGGTAAAGCCCATCCCCAACAAACGTATTGCAACCAGTATCAACAAGCACGGTTTCTCCTGGAAGAATCTTTGGCGACATTGAATTACCGGTTCCGGTAATCAGCTTGAGTCGGCCTGGTGGTGGTAAGAATCCAATGAGTGAGCGGATGTAATTTGGTGGGAACTCAACAGAGTTTATAACTTCAGGTAAATCCTCATTGATACGACCTTGTCCCATATGTGCTTCCGCTTCAATATGATCAACGCGGATATAATTGTTCCCAGTTTTAATGTTAAATGTTGATGATTTCTGTTTCTCAGGAATCTCCTCTTCTGTGGTGTCTAAATAGTTATCTGGCATACCTGCTAACTTTTCGATAGTCCGTGCTTTTTTCTCGCCGAATGACTTTTTCCCATTGAGTAGTCCGGAAAGTTCTCCCTGATTGATACTAATTTTTTCTACGAAAAAAGCTTGCACACCGTCGTGCTGATCTTGAATCCATTGTTTTAGGCGACGTCTACGTAAGGCAACGGTAGCAGAATTAAGAAATGTCATTGCTTTATTTTCACTTACCGATTGGTAATTTACCAAAGGGTATTGACTTGTAATTATCATATGGTAATTTGAGTGCATGGAAACCCTGCGTACCTACCTCGCGACACTCATCCCAGCCGAACAGGCGCACTACGCTCGTAAAGCGAATACCACCATTGGTTACCTGCGAAAGGCTCTCTGCAAGGGACAGCGATTTGATGGTGCACTGGCGCGACGCCTTGATGAAGCCAGTGGTGGCCGTGTTTCTCGTTACGATCTTCGCCCAGACGTATTCGGCGCTCCCCCCACAGGCCACAGGCAGGAGGTGTCCGATGCGGCGTGAGAAAGGCACTTTTTTTACTTCGCTGTGGGGTGCTTCTTTACGT |
| 11 | FX0.5K05228 | TAGTCCGGAAAGTTCTCCCTGATTGATACTAATTTTTTCTACGAAAAAAGCTTGCACACCGTCGTGCTGATCTTGAATCCATTGTTTTAGGCGACGTCTACGTAAGGCAACGGTAGCAGAATTAAGAAATGTCATTGCTTTATTTTCACTTACCGATTGGTAATTTACCAAAGGGTATTGACTTGTAATTATCATATGGTAATTTGAGTGCATGGAAACCCTGCGTACCTACCTCGCGACACTCATCCCAGCCGAACAGGCGCACTACGCTCGTAAAGCGAATACCACCATTGGTTACCTGCGAAAGGCTCTCTGCAAGGGACAGCGATTTGATGGTGCACTGGCGCGACGCCTTGATGAAGCCAGTGGTGGCCGTGTTTCTCGTTACGATCTTCGCCCAGACGTATTCGGCGCTCCCCCCACAGGCCACAGGCAGGAGGTGTCCGATGCGGCGTGAGAAAGGCACTTTTTTTACTTCGCTGTGGGGTGCTTCTTTACGT |
| 12 | FX0.5K05115 | CGCTTCGCGCTCTTTTCTGACTCTGCTACCGATAGTTTCCATAATTAGGAATCCTCACAGAGTTCTGTAGCAGGATGCTGTTGTTTTCCACTACAGCATCCTGTAGATTGTTGCCCCATGGACTGGAAAGCACGAATTAAGCTCTTGATTGATGGTGGTGCGACGGTTGATTCCATCGCCGCCCATATGGGCGTCACGCCTAACGCGGTGCGTGAAATTTTGGCTGGGAGAACTAAATCCCCGCGCGCTGATGCCGCTTTCCGCCTCGCCTCACTCATGCCGGACTCTCCCCCCACAGGCCACAGGCAGGAGGTGTCCAATGCGGCGTGAAGGATTTCTCGATCTAGCTGATTCCAGCCTGGAGCCTTCGTTAGGAGGGAGCAACCTGCTCATTGTGAGAGTCCCATTCGCTGGTCCTCCCAGCGTACCGACATTCTTTTGGACGGAAATATCCGGACCGGAGAATAAAAAGTTGGCGTATATCAAGTTTGAGCGGCGGC |
| 13 | FX0.8K00387 | ACTATATATTACGCTTAGTGTAATAGTGGTATATTAATCTTATTAAGCAGCATGGAAGTCAAGTTTGAATATCCATCGTTGGAGCGAATGGAAACAAACCAGAAATACACTGCTGGCTTGGTCAAGGCGTTCCACCGGCGCATATAGTTCGTCAGGGCATCGACTGACGAACGTGCGTTCAGAGCCACGACTGACGAACGTGCGTTCTATGCCATGAAATCACTGCACTACAAAAAAACTCAAAGATGATCCGGGCAACCTGTACTCCATGCACCTAAACGACCAATAGCACCTGATCATGTACCTAAAAGCAAAAGAAGATGACACAAGAAATCTTGTCGTGATTGTCTCAATTGTCGATTATTACTGAACTCAAGAGCACGCCATGAACACGTTCGCCGAAGTTTTCCCACCAGGCGAGTTCCTCCGCGATGAACTCGAGGCGCGTCATTGGACGCAAACTGAACTGGCCGAAATCATCGGTCGTCCGGTACACACGATTAACGAGATCATTGCAGGCAAGAAGGCTATTACTCCGGAGACAGCCATCCAGTTGGGTAAATCGCTGGGGACAGGTCCAGAAGTCTGGATGAATCTTGAAAGCCAGTACCAGCTCTCCAAAGTCAGCGGCGTTAATTAACGGTTGCTTACAGTTACATTGATGCAGAAAGGATTCTGGAGAATTGTTTTCCCTGTGCCATTACGTGAAACACCTGTCGCTGCATATTTATAAAAGTGATGTACTTCACACACTCACAAGAGAAAATTGATATGAGCCAATTCAATTCTTTGGAAATGTG |
| 14 | FX0.8K03197 | TCTGTTGCAATAGATTTTGTGGCGGTTGTGGCTTCGATTTTCTCGCCTTTTCCTGTTTCCAGCCATCGCAAAGAAACGCCCAACGCATCAGCAATAAGTCGAAGCTTTGTAGTCGTTTGCATTCCACCACGTTCCAATTCAGCAATTGTGCTGTAGCCGATCCCTGTTTTCTTGGCGAAATCGGATCTGTTGATGTTCTGCGCTTCGCGCTCTTTTCTGACTCTGCTACCGATAGTTTCCATAATTAGGAATCCTCACAGAGTTCTGTAGCAGGATGCTGTTGTTTTCCACTACAGCATCCTGTAGATTGTTGCCCCATGGACTGGAAAGCACGAATTAAGCTCTTGATTGATGGTGGTGCGACGGTTGATTCCATCGCCGCCCATATGGGCGTCACGCCTAACGCGGTGCGTGAAATTTTGGCTGGGAGAACTAAATCCCCGCGCGCTGATGCCGCTTTCCGCCTCGCCTCACTCATGCCGGACTCTCCCCCCACAGGCCACAGGCAGGAGGTGTCCAATGCGGCGTGAAGGATTTCTCGATCTAGCTGATTCCAGCCTGGAGCCTTCGTTAGGAGGGAGCAACCTGCTCATTGTGAGAGTCCCATTCGCTGGTCCTCCCAGCGTACCGACATTCTTTTGGACGGAAATATCCGGACCGGAGAATAAAAAGTTGGCGTATATCAAGTTTGAGCGGCGGCTTTTCATAAGGTCGGAATCACTGTTTGTAGCCACCGAATGGCGTCCGGCAGATTCCGGAGACTTGCGTCCAGAACCTTTTCTGCGATTTTCTGGATACCA |
| 15 | FX0.3K08525 | TAACGCGGTGCGTGAAATTTTGGCTGGGAGAACTAAATCCCCGCGCGCTGATGCCGCTTTCCGCCTCGCCTCACTCATGCCGGACTCTCCCCCCACAGGCCACAGGCAGGAGGTGTCCAATGCGGCGTGAAGGATTTCTCGATCTAGCTGATTCCAGCCTGGAGCCTTCGTTAGGAGGGAGCAACCTGCTCATTGTGAGAGTCCCATTCGCTGGTCCTCCCAGCGTACCGACATTCTTTTGGACGGAAATATCCGGACCGGAGAATAAAAAGTTGGCGTATATCAAGTTTGAGCGGCGGC |
| 16 | FX0.3K08743 | AGGCATGCACCGCAGTGGTGAATACACGTCGACCAGGCTCCAGCTTAACGATATTGGAGGTCAGCACGGTGCTCACGTGTCGACGGAACTTGTCCCAGCGCAGGCCATGCACCGCCAGTGGTGCAATACACGTCGCACCTGCCAGGCCATGCCATGCCATGCTCGCGGTACTGGCAGGCATCCTCTCAGCAGTCGACCCACGAGCTCCAACGCTTAACCGACTAATTTAGAGGACGAGTCACGCACCGGGTGCTCACCGTGTCGACCGAGACACTTGTACCACGGGAACGGCACTGCACC |
| 17 | FX0.3K08523 | CTGGAATCATGGAATCACCACAACCAGTTATTAGTTTCAGCCTTCCCTTCTGGGGTACGAAGCCAATTAAGCTGCGAATGAATGCCTCAGTATATTCCACCGATCGGATGACATCTGGGTAATCTAGATTCACGCGGCTCTCACCCATACCTGCCTCCGCATCTAAATGTTCAACGCGGATATAATGTTGCGATATTTTTTCTGTTGCAATAGATTTTGTGGCGGTTGTGGCTTCGATTTTCTCGCCTTTTCCTGTTTCCAGCCATCGCAAAGAAACGCCCAACGCATCAGCAATAAGTC |
| 18 | FX0.5K05227 | ACGAAGTCAGGATAAAGAGCTTTATCCATGCTGGTAATGTATATGTATCCGTTTCTATCATGTAATGCTTTTATTTGTTGTCCGTTACCTGTATTGACTAGGTAAAGCCCATCCCCAACAAACGTATTGCAACCAGTATCAACAAGCACGGTTTCTCCTGGAAGAATCTTTGGCGACATTGAATTACCGGTTCCGGTAATCAGCTTGAGTCGGCCTGGTGGTGGTAAGAATCCAATGAGTGAGCGGATGTAATTTGGTGGGAACTCAACAGAGTTTATAACTTCAGGTAAATCCTCATTGATACGACCTTGTCCCATATGTGCTTCCGCTTCAATATGATCAACGCGGATATAATTGTTCCCAGTTTTAATGTTAAATGTTGATGATTTCTGTTTCTCAGGAATCTCCTCTTCTGTGGTGTCTAAATAGTTATCTGGCATACCTGCTAACTTTTCGATAGTCCGTGCTTTTTTCTCGCCGAATGACTTTTTCCCATTGAG |
| 19 | FX0.3K05489 | AATGTTTTAGAGAATAAGGGATGTGTTGACAATAGATACACAATAAATTTACGATGTGTATCTACTGTAAATACAATCGGAGAGGGCGATGCGCGACGCTTCCATCAACTTACGTGCCATGCCTGAGCAGCGCGACTTGATTGACCAGGCTGCTAATCTATTAGGCAAGAATCGCTCAGACTTCATGCTAGAAGCTGCGTGCGAGCGTGCGAAAGCAATTATCCTGGATCAAGTCTTTTTCAATCTGGACGAAGAGAGGTTCCGGCAATTCACTGTGTTGCTTAATGCGCCACAAGACAC |
| 20 | FX0.5K03294 | TCCATCAACTTACGTGCCATGCCTGAGCAGCGCGACTTGATTGACCAGGCTGCTAATCTATTAGGCAAGAATCGCTCAGACTTCATGCTAGAAGCTGCGTGCGAGCGTGCGAAAGCAATTATCCTGGATCAAGTCTTTTTCAATCTGGACGAAGAGAGGTTCCGGCAATTCACTGTGTTGCTTAATGCGCCACAAGACACCAATCCAGGCCTTAATCGCCTGATGGCAATTAAGGCTCCGTGGAGCATCAGCAATACGTGAATATGCAGATTTCGACACCTCATTCACTCACGGCAGCGCATCGACTTGATGAATTCAATTGCGGTGAGCCATCACTGGATGATTGGCTTAAACGACGCGCACTGACCAACCATTTAAACGGGGCCAGCCGGACCTTCGTGGTTATCGATGCCAACCAATACGTGCTTGGGTACTACGCTTTGGCAGCCGGGGCTGTAGCGCATCAAGAGGCCACCCGTGCTATCCGACGTAACATGCCT |
| 21 | FX0.3K08524 | GAAGCTTTGTAGTCGTTTGCATTCCACCACGTTCCAATTCAGCAATTGTGCTGTAGCCGATCCCTGTTTTCTTGGCGAAATCGGATCTGTTGATGTTCTGCGCTTCGCGCTCTTTTCTGACTCTGCTACCGATAGTTTCCATAATTAGGAATCCTCACAGAGTTCTGTAGCAGGATGCTGTTGTTTTCCACTACAGCATCCTGTAGATTGTTGCCCCATGGACTGGAAAGCACGAATTAAGCTCTTGATTGATGGTGGTGCGACGGTTGATTCCATCGCCGCCCATATGGGCGTCACGCC |
| 22 | FX0.3K08522 | GCAGCCACCGCCGCAAGCGGCTAAGCAGCCTTAATCATCACTATTTTTATAAATGGTTGAGTAGTTTTTAATTAAACCGTTCGATTTTGTTTTTAATGTAAATCTTCCCACCGATAAAACCCTCGCTAGGGAACGGGATCGGTTGGTAGAGGGGATTTGCGCTCACCACATACACGGCGTCGCCACGATCTTGTAACGCCTTGATCTGCTGGCCATGCCCAATATTAATGAGGTAAATGCCGTCCCCATCAAACGACTGAATACCCGTATCAACGAGCACGACTTCACCAGGCTGGATTA |
