# Supplementary material for: PrimerSNP: a web tool for whole-genome selection of allele-specific and common primers of phylogenetically-related bacterial genomic sequences
Source: BMC Microbiol. 2008 Oct 20;8:185. doi: 10.1186/1471-2180-8-185 (PMC2579435; doi:10.1186/1471-2180-8-185)
Supplement: Additional file 2 — The result of experimental validation of the specific primers designed for 9a5c , Temecula1 and Dixon Strains of Xylella fastidiosa. The data provided the experimental validation of the specific primers that are designed using PrimerSNP. [file 1471-2180-8-185-S2.doc]

Supplementary Table 2 The Result of Experimental Validation of the Specific Primers Designed for *9a5c*, *Temecula1* and *Dixon* Strains of *Xylella* *fastidiosa* (“+” means PCR positive, “-” means PCR negative)

| No. | Strain | Gene | Weight | ΔG° | | | DNA template | | |
| --- | --- | --- | --- | --- | --- | --- | --- | --- | --- |
|  |  |  |  | Left | Right | Total | 9a5c | Temecula-1 | Dixon |
| 1 | 9a5c | XF2039 | 63462 | -27.5 | -29.3 | -56.8 | + | - | - |
| 2 | 9a5c | XF1774 | 59574 | -28.1 | -27.8 | -55.9 | + | - | - |
| 3 | 9a5c | XF0328 | 57709 | -28.7 | -28.4 | -57.1 | + | - | - |
| 4 | 9a5c | XF1761 | 57209 | -27.7 | -27.7 | -55.4 | + | - | - |
| 5 | 9a5c | XF1719 | 56679 | -28.9 | -27 | -55.9 | + | - | - |
| 6 | 9a5c | XF2741 | 55710 | -27.1 | -27.5 | -54.6 | + | - | - |
| 7 | 9a5c | XF1781 | 55644 | -28.5 | -27.6 | -56.1 | + | + | - |
| 8 | 9a5c | XF2294 | 54723 | -27.7 | -27 | -54.7 | + | - | - |
| 9 | 9a5c | XF1770 | 53960 | -27.5 | -28.3 | -55.8 | + | - | - |
| 10 | 9a5c | XF2745 | 53241 | -26.8 | -27.8 | -54.6 | + | + | + |
| 11 | 9a5c | XF1733 | 52946 | -28.1 | -26.4 | -54.5 | + | - | + |
| 12 | 9a5c | XF2584 | 52780 | -26.0 | -27.5 | -53.5 | + | - | - |
| 13 | 9a5c | XF1755 | 52687 | -27.2 | -26.9 | -54.1 | + | + | - |
| 14 | 9a5c | XF0689 | 52547 | -29.0 | -28.0 | -57.0 | + | - | - |
| 15 | 9a5c | XF0779 | 51805 | -27.6 | -26.2 | -53.8 | + | - | - |
| 16 | 9a5c | XF1384 | 51408 | -27.7 | -29.1 | -56.8 | + | + | - |
| 17 | 9a5c | XF0696 | 51270 | -26.2 | -27.1 | -53.3 | + | - | - |
| 18 | 9a5c | XF0897 | 51107 | -28.8 | -28.9 | -57.7 | + | - | - |
| 19 | 9a5c | XF2037 | 51069 | -26.0 | -28.7 | -54.7 | + | - | - |
| 20 | 9a5c | XF2715 | 50861 | -26.8 | -28.0 | -54.8 | + | - | - |
| 21 | 9a5c | XF1743 | 50683 | -27.6 | -27.6 | -55.2 | + | - | - |
| 22 | 9a5c | XF1727 | 44085 | -28.6 | -27.6 | -56.2 | + | - | - |
| 23 | 9a5c | XF0496 | 39350 | -27.2 | -29.0 | -56.2 | + | - | + |
| 24 | 9a5c | XF1772 | 36815 | -26.9 | -28.1 | -55.0 | + | - | - |
| 25 | 9a5c | XF0491 | 33424 | -27.3 | -25.6 | -52.9 | + | - | - |
| 26 | 9a5c | XF0935 | 32486 | -26.6 | -25.7 | -52.3 | + | - | - |
| 27 | 9a5c | XF1008 | 30497 | -25.6 | -26.7 | -52.3 | + | + | - |
| 28 | 9a5c | XF1437 | 28676 | -27.2 | -26.6 | -53.8 | + | - | - |
| 29 | 9a5c | XF0693 | 26624 | -28.9 | -27.3 | -56.2 | + | - | - |
| 30 | 9a5c | XF1358 | 24864 | -28.2 | -27.8 | -56.0 | + | - | - |
| 31 | 9a5c | XF1815 | 24608 | -26.2 | -26.4 | -52.6 | + | - | - |
| 32 | 9a5c | XF0323 | 22593 | -27.4 | -28.9 | -56.3 | + | - | - |
| 33 | 9a5c | XF2091 | 20616 | -27.5 | -28.5 | -56.0 | + | - | - |
| 34 | 9a5c | XF1647 | 19267 | -29.1 | -27.6 | -56.7 | + | - | - |
| 35 | 9a5c | XF2759 | 18560 | -26.7 | -28.0 | -54.7 | + | - | - |
| 36 | 9a5c | XF0486 | 18392 | -25.8 | -26.4 | -52.2 | + | - | - |
| 37 | 9a5c | XF1785 | 17226 | -25.5 | -27.1 | -52.6 | + | - | - |
| 38 | 9a5c | XF0883 | 16649 | -27.2 | -26.6 | -53.8 | + | - | + |
| 39 | 9a5c | XF0321 | 16512 | -28.0 | -27.9 | -55.9 | + | + | + |
| 40 | 9a5c | XF2426 | 16420 | -28.2 | -28.0 | -56.2 | + | + | - |
| 41 | 9a5c | XF0595 | 16393 | -27.1 | -27.5 | -54.6 | + | + | + |
| 1 | Temecula1 | PD1608 | 54247 | -28.7 | -28.3 | -57.0 | - | + | - |
| 2 | Temecula1 | PD2097 | 50439 | -27.4 | -27.9 | -55.3 | - | + | - |
| 3 | Temecula1 | PD2071 | 49017 | -27.6 | -27.8 | -55.4 | - | + | - |
| 4 | Temecula1 | PD0304 | 48542 | -25.0 | -29.1 | -54.1 | - | + | - |
| 5 | Temecula1 | PD2075 | 46371 | -27.8 | -30.4 | -58.2 | - | + | - |
| 6 | Temecula1 | PD0833 | 44121 | -26.9 | -27.0 | -53.9 | - | + | - |
| 7 | Temecula1 | PD1507 | 43520 | -27.3 | -26.7 | -54.0 | - | + | - |
| 8 | Temecula1 | PD1607 | 43235 | -27.6 | -28.2 | -55.8 | - | + | - |
| 9 | Temecula1 | PD0088 | 36128 | -27.9 | -27.0 | -54.9 | - | + | - |
| 10 | Temecula1 | PD0087 | 35906 | -28.4 | -27.9 | -56.3 | - | + | - |
| 11 | Temecula1 | PD1014 | 33797 | -25.8 | -26.6 | -52.4 | - | + | - |
| 12 | Temecula1 | PD0920 | 31110 | -28.2 | -26.5 | -54.7 | - | + | - |
| 13 | Temecula1 | PD1190 | 30783 | -26.8 | -26.7 | -53.5 | - | + | + |
| 14 | Temecula1 | PD2094 | 30097 | -28.6 | -25.4 | -54.0 | - | + | + |
| 15 | Temecula1 | PD1283 | 28932 | -28.2 | -27.3 | -55.5 | - | + | - |
| 16 | Temecula1 | PD1242 | 28868 | -27.4 | -27.7 | -55.1 | - | + | - |
| 17 | Temecula1 | PD0305 | 27440 | -27.9 | -26.5 | -54.4 | - | + | - |
| 18 | Temecula1 | PD1349 | 27065 | -26.5 | -27.5 | -54.0 | - | + | + |
| 19 | Temecula1 | PD1951 | 26645 | -30.0 | -28.3 | -58.3 | - | + | - |
| 20 | Temecula1 | PD1506 | 26294 | -28.0 | -27.5 | -55.5 | - | + | - |
| 21 | Temecula1 | PD2109 | 25160 | -25.0 | -28.4 | -53.4 | - | + | - |
| 22 | Temecula1 | PD1434 | 25034 | -27.9 | -29.7 | -57.6 | - | + | - |
| 23 | Temecula1 | PD1924 | 24644 | -28.4 | -26.1 | -54.5 | - | + | - |
| 24 | Temecula1 | PD1002 | 24644 | -27.7 | -25.1 | -52.8 | - | + | - |
| 25 | Temecula1 | PD1416 | 21058 | -28.3 | -26.0 | -54.3 | - | + | - |
| 26 | Temecula1 | PD2108 | 21025 | -27.6 | -28.9 | -56.5 | + | + | - |
| 27 | Temecula1 | PD1362 | 20481 | -27.9 | -29.8 | -57.7 | - | + | - |
| 28 | Temecula1 | PD0502 | 17440 | -26.8 | -28.4 | -55.2 | - | + | - |
| 29 | Temecula1 | PD1136 | 17132 | -26.5 | -26.4 | -52.9 | - | + | - |
| 30 | Temecula1 | PD2030 | 16804 | -27.1 | -26.4 | -53.5 | - | + | - |
| 31 | Temecula1 | PD0996 | 16768 | -27.4 | -26.5 | -53.9 | - | + | - |
| 32 | Temecula1 | PD1323 | 16768 | -27.4 | -25.7 | -53.1 | - | + | - |
| 33 | Temecula1 | PD0980 | 16708 | -29.5 | -27.7 | -57.2 | - | + | - |
| 34 | Temecula1 | PD1107 | 16640 | -27.1 | -27.7 | -54.8 | - | + | - |
| 35 | Temecula1 | PD1125 | 16514 | -27.2 | -27.5 | -54.7 | - | + | - |
| 36 | Temecula1 | PD0579 | 16456 | -28.1 | -26.1 | -54.2 | - | + | - |
| 37 | Temecula1 | PD2080 | 16448 | -26.0 | -27.2 | -53.2 | - | + | - |
| 38 | Temecula1 | PD1240 | 16420 | -26.4 | -27.5 | -53.9 | - | + | - |
| 39 | Temecula1 | PD0993 | 16416 | -28.5 | -27.2 | -55.7 | - | + | - |
| 40 | Temecula1 | PD1223 | 16416 | -24.7 | -26.8 | -51.5 | - | + | - |
| 41 | Temecula1 | PD0478 | 16400 | -29.5 | -27.1 | -56.6 | + | + | + |
| 42 | Temecula1 | PD0960 | 16393 | -27.2 | -27.9 | -55.1 | - | + | - |
| 43 | Temecula1 | PD1451 | 16392 | -29.3 | -28.1 | -57.4 | + | + | + |
| 44 | Temecula1 | PD0817 | 16384 | -26.3 | -29.9 | -56.2 | + | + | + |
| 1 | Dixon | FX0.3K08522 | 58432 | -28.1 | -27.6 | -55.7 | - | - | + |
| 2 | Dixon | FX0.3K08524 | 53455 | -28.3 | -26.7 | -55.0 | - | - | + |
| 3 | Dixon | FX0.5K03294 | 49262 | -27.1 | -26.2 | -53.3 | + | + | + |
| 4 | Dixon | FX0.3K05489 | 41715 | -27.4 | -27.4 | -54.8 | + | + | + |
| 5 | Dixon | FX0.5K05227 | 37617 | -26.7 | -25.2 | -51.9 | - | - | + |
| 6 | Dixon | FX0.3K08523 | 34468 | -27.3 | -26.8 | -54.1 | - | - | + |
| 7 | Dixon | FX0.3K08743 | 33558 | -28.4 | -29.5 | -57.9 | - | - | - |
| 8 | Dixon | FX0.3K08525 | 31284 | -27.8 | -27.1 | -54.9 | - | - | + |
| 9 | Dixon | FX0.8K03197 | 30693 | -28.3 | -26.1 | -54.4 | - | - | + |
| 10 | Dixon | FX0.8K00387 | 29559 | -27.0 | -27.0 | -54.0 | - | - | - |
| 11 | Dixon | FX0.5K05115 | 29148 | -26.5 | -27.1 | -53.6 | - | - | + |
| 12 | Dixon | FX0.5K05228 | 28686 | -30.5 | -29.7 | -60.2 | - | - | + |
| 13 | Dixon | FX1K02614 | 26787 | -27.1 | -27.8 | -54.9 | - | - | + |
| 14 | Dixon | FX0.5K05114 | 24914 | -25.6 | -26.8 | -52.4 | - | - | + |
| 15 | Dixon | FX2K00754 | 22912 | -25.8 | -27.1 | -52.9 | - | - | + |
| 16 | Dixon | FX0.8K01884 | 22608 | -27.8 | -27.3 | -55.1 | - | - | + |
| 17 | Dixon | FX0.5K05247 | 18505 | -28.8 | -28.6 | -57.4 | - | - | - |
| 18 | Dixon | FX0.5K03872 | 18289 | -25.1 | -27.4 | -52.5 | - | + | + |
| 19 | Dixon | FX0.8K03268 | 17905 | -28.1 | -30.0 | -58.1 | - | - | + |
| 20 | Dixon | FX0.5K03514 | 16964 | -26.2 | -26.7 | -52.9 | - | - | + |
| 21 | Dixon | FX0.5K03011 | 16672 | -26.9 | -27.7 | -54.6 | - | + | + |
| 22 | Dixon | FX0.3K05203 | 16400 | -27.0 | -27.4 | -54.4 | + | + | + |

**Primers and PCR Kits**

All primers and PCR kits were obtained from Invitrogen (Carlsbad, CA). Genomic DNA of three strains (9a5c, Temecula1, Dixon and Ann1) of the bacterium *Xylella fastidiosa* was used as template for validation of the specific primers generated by PrimerSNP. The genomic DNA 9a5c was obtained from Brazil and genomic DNA from the other strains were prepared as previously described [6]. The PCR protocol was 35 cycles of each of the following: 95ºC for 30 s, 60ºC for 90 s and 72ºC for 60 s.
