# Supplementary material for: PrimerSNP: a web tool for whole-genome selection of allele-specific and common primers of phylogenetically-related bacterial genomic sequences
Source: BMC Microbiol. 2008 Oct 20;8:185. doi: 10.1186/1471-2180-8-185 (PMC2579435; doi:10.1186/1471-2180-8-185)
Supplement: Additional file 3 — t-test on the effect of weight score and free energy (ΔG°) on the specificity of primers. The data provided the statistical analysis of the primers' specificity of two groups' primers that have different values of weight score and free energy (ΔG°). [file 1471-2180-8-185-S3.doc]

Supplementary Table 3

*t*-test on the effect of weight score and free energy (ΔG°) on the specificity of primers

Welch Two Sample t-test

data: good and bad

t = 2.0044, df = 89.213, p-value = 0.04806

alternative hypothesis: true difference in means is not equal to 0

95 percent confidence interval:

0.001407444 0.320814778

sample estimates:

mean of x mean of y

0.8611111 0.7000000

No. Strain Gene Weight leftΔG° rightΔG° totalΔG° specificity

1 9a5c XF2039 63462 -27.5 -29.3 -56.8 1

2 9a5c XF1774 59574 -28.1 -27.8 -55.9 1

3 9a5c XF0328 57709 -28.7 -28.4 -57.1 1

4 9a5c XF1761 57209 -27.7 -27.7 -55.4 1

5 9a5c XF1719 56679 -28.9 -27 -55.9 1

6 9a5c XF2741 55710 -27.1 -27.5 -54.6 1

7 9a5c XF1781 55644 -28.5 -27.6 -56.1 0

8 9a5c XF2294 54723 -27.7 -27 -54.7 1

9 9a5c XF1770 53960 -27.5 -28.3 -55.8 1

10 9a5c XF2745 53241 -26.8 -27.8 -54.6 0

11 9a5c XF1733 52946 -28.1 -26.4 -54.5 0

12 9a5c XF2584 52780 -26.0 -27.5 -53.5 1

13 9a5c XF1755 52687 -27.2 -26.9 -54.1 0

14 9a5c XF0689 52547 -29.0 -28.0 -57.0 1

15 9a5c XF0779 51805 -27.6 -26.2 -53.8 1

16 9a5c XF1384 51408 -27.7 -29.1 -56.8 0

17 9a5c XF0696 51270 -26.2 -27.1 -53.3 1

18 9a5c XF0897 51107 -28.8 -28.9 -57.7 1

19 9a5c XF2037 51069 -26.0 -28.7 -54.7 1

20 9a5c XF2715 50861 -26.8 -28.0 -54.8 1

21 9a5c XF1743 50683 -27.6 -27.6 -55.2 1

22 9a5c XF1727 44085 -28.6 -27.6 -56.2 1

23 9a5c XF0496 39350 -27.2 -29.0 -56.2 0

24 9a5c XF1772 36815 -26.9 -28.1 -55.0 1

25 9a5c XF0491 33424 -27.3 -25.6 -52.9 1

26 9a5c XF0935 32486 -26.6 -25.7 -52.3 1

27 9a5c XF1008 30497 -25.6 -26.7 -52.3 0

28 9a5c XF1437 28676 -27.2 -26.6 -53.8 1

29 9a5c XF0693 26624 -28.9 -27.3 -56.2 1

30 9a5c XF1358 24864 -28.2 -27.8 -56.0 1

31 9a5c XF1815 24608 -26.2 -26.4 -52.6 1

32 9a5c XF0323 22593 -27.4 -28.9 -56.3 1

33 9a5c XF2091 20616 -27.5 -28.5 -56.0 1

34 9a5c XF1647 19267 -29.1 -27.6 -56.7 1

35 9a5c XF2759 18560 -26.7 -28.0 -54.7 1

36 9a5c XF0486 18392 -25.8 -26.4 -52.2 1

37 9a5c XF1785 17226 -25.5 -27.1 -52.6 1

38 9a5c XF0883 16649 -27.2 -26.6 -53.8 0

39 9a5c XF0321 16512 -28.0 -27.9 -55.9 0

40 9a5c XF2426 16420 -28.2 -28.0 -56.2 0

41 9a5c XF0595 16393 -27.1 -27.5 -54.6 0

1 Temecula1 PD1608 54247 -28.7 -28.3 -57.0 1

2 Temecula1 PD2097 50439 -27.4 -27.9 -55.3 1

3 Temecula1 PD2071 49017 -27.6 -27.8 -55.4 1

4 Temecula1 PD0304 48542 -25.0 -29.1 -54.1 1

5 Temecula1 PD2075 46371 -27.8 -30.4 -58.2 1

6 Temecula1 PD0833 44121 -26.9 -27.0 -53.9 1

7 Temecula1 PD1507 43520 -27.3 -26.7 -54.0 1

8 Temecula1 PD1607 43235 -27.6 -28.2 -55.8 1

9 Temecula1 PD0088 36128 -27.9 -27.0 -54.9 1

10 Temecula1 PD0087 35906 -28.4 -27.9 -56.3 1

11 Temecula1 PD1014 33797 -25.8 -26.6 -52.4 1

12 Temecula1 PD0920 31110 -28.2 -26.5 -54.7 1

13 Temecula1 PD1190 30783 -26.8 -26.7 -53.5 0

14 Temecula1 PD2094 30097 -28.6 -25.4 -54.0 0

15 Temecula1 PD1283 28932 -28.2 -27.3 -55.5 1

16 Temecula1 PD1242 28868 -27.4 -27.7 -55.1 1

17 Temecula1 PD0305 27440 -27.9 -26.5 -54.4 1

18 Temecula1 PD1349 27065 -26.5 -27.5 -54.0 0

19 Temecula1 PD1951 26645 -30.0 -28.3 -58.3 1

20 Temecula1 PD1506 26294 -28.0 -27.5 -55.5 1

21 Temecula1 PD2109 25160 -25.0 -28.4 -53.4 1

22 Temecula1 PD1434 25034 -27.9 -29.7 -57.6 1

23 Temecula1 PD1924 24644 -28.4 -26.1 -54.5 1

24 Temecula1 PD1002 24644 -27.7 -25.1 -52.8 1

25 Temecula1 PD1416 21058 -28.3 -26.0 -54.3 1

26 Temecula1 PD2108 21025 -27.6 -28.9 -56.5 0

27 Temecula1 PD1362 20481 -27.9 -29.8 -57.7 1

28 Temecula1 PD0502 17440 -26.8 -28.4 -55.2 1

29 Temecula1 PD1136 17132 -26.5 -26.4 -52.9 1

30 Temecula1 PD2030 16804 -27.1 -26.4 -53.5 1

31 Temecula1 PD0996 16768 -27.4 -26.5 -53.9 1

32 Temecula1 PD1323 16768 -27.4 -25.7 -53.1 1

33 Temecula1 PD0980 16708 -29.5 -27.7 -57.2 1

34 Temecula1 PD1107 16640 -27.1 -27.7 -54.8 1

35 Temecula1 PD1125 16514 -27.2 -27.5 -54.7 1

36 Temecula1 PD0579 16456 -28.1 -26.1 -54.2 1

37 Temecula1 PD2080 16448 -26.0 -27.2 -53.2 1

38 Temecula1 PD1240 16420 -26.4 -27.5 -53.9 1

39 Temecula1 PD0993 16416 -28.5 -27.2 -55.7 1

40 Temecula1 PD1223 16416 -24.7 -26.8 -51.5 1

41 Temecula1 PD0478 16400 -29.5 -27.1 -56.6 0

42 Temecula1 PD0960 16393 -27.2 -27.9 -55.1 1

43 Temecula1 PD1451 16392 -29.3 -28.1 -57.4 0

44 Temecula1 PD0817 16384 -26.3 -29.9 -56.2 0

1 Dixon FX0.3K08522 58432 -28.1 -27.6 -55.7 1

2 Dixon FX0.3K08524 53455 -28.3 -26.7 -55.0 1

3 Dixon FX0.5K03294 49262 -27.1 -26.2 -53.3 0

4 Dixon FX0.3K05489 41715 -27.4 -27.4 -54.8 0

5 Dixon FX0.5K05227 37617 -26.7 -25.2 -51.9 1

6 Dixon FX0.3K08523 34468 -27.3 -26.8 -54.1 1

7 Dixon FX0.3K08743 33558 -28.4 -29.5 -57.9 0

8 Dixon FX0.3K08525 31284 -27.8 -27.1 -54.9 1

9 Dixon FX0.8K03197 30693 -28.3 -26.1 -54.4 1

10 Dixon FX0.8K00387 29559 -27.0 -27.0 -54.0 0

11 Dixon FX0.5K05115 29148 -26.5 -27.1 -53.6 1

12 Dixon FX0.5K05228 28686 -30.5 -29.7 -60.2 1

13 Dixon FX1K02614 26787 -27.1 -27.8 -54.9 1

14 Dixon FX0.5K05114 24914 -25.6 -26.8 -52.4 1

15 Dixon FX2K00754 22912 -25.8 -27.1 -52.9 1

16 Dixon FX0.8K01884 22608 -27.8 -27.3 -55.1 1

17 Dixon FX0.5K05247 18505 -28.8 -28.6 -57.4 0

18 Dixon FX0.5K03872 18289 -25.1 -27.4 -52.5 0

19 Dixon FX0.5K03514 16964 -26.2 -26.7 -52.9 1

20 Dixon FX0.5K03011 16672 -26.9 -27.7 -54.6 0

21 Dixon FX0.3K05203 16400 -27.0 -27.4 -54.4 0
